# Richang Cao: pioneer advocate of dialectical materialism applied to psychological research

**DOI:** 10.1007/s13238-019-0615-2

**Published:** 2019-03-12

**Authors:** Baoyuan Zhang

**Affiliations:** Beijing Institutes of Life Science, Chinese Academy of Sciences, Beijing, 100101 China

“Don’t tell me what for or for what, just tell me what I should do!” It is an urgent demand that we always heard in our labs. However, the results end up being far from satisfaction when postgraduate do experiments strictly by their adviser’s guidance. There are many students who are completely indifferent to the theoretical hypothesis and concluding analysis of their research; even if they have read a lot of literature and repeated a lot of experiments in their daily routine. When we ask about theoretical foundations of their research, they are at a loss and can’t answer at all.

**Figure 1:**
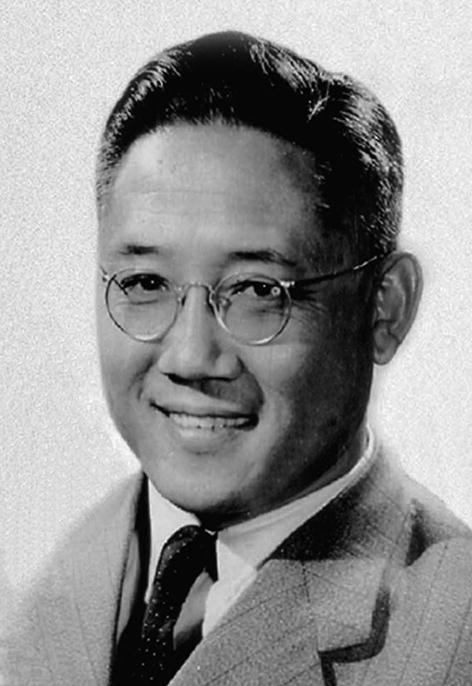
**Prof. Richang Cao (Jih-chang Tsao)**

Though there are many reasons for these disappointments, the most important one is because we are used to accepting or following tradition—what other people did—and rarely attempt to explore what other people have never done before. Confucius said: “Learning without thinking leads to confusion; thinking without learning ends in danger”. Speculation is a vital part of doing research, like psychologist Richang Cao (Jih-chang Tsao, 曹日昌), a pioneer advocate for applied dialectical materialism to psychological research, reviews his work and methodology which is still worth learning and using today (Zhang, 2006) (Fig. 1).

Prof. Cao is one of the main founders of the construction and development of psychological science after the founding of new China. He advocated that psychology should be used to study the universal law of psychological phenomena, depending on social practice, while guided by the epistemology of Marxism. He explained the development of psychological phenomena and psychology with three universal principles which are the law of unity of opposites, the law of qualitative and quantitative mutual change, and the law of negation of negation. These laws play an extremely important role in the development of psychology in China.

In 1943, Prof. Cao started to use material dialectics to analyze some important questions of psychological testing, and published *Several Questions of Principle of Psychological Test*. In his article, he analyzed the inadequacies of the existing psychological tests by a few common categories of dialectical relationships including: quality and quantity, parts and whole, surface and essence, and so on. He clearly highlighted limitations of the existing tests, like they did only pay attention to the number of repetitions but didn’t care about the quality of the analysis, only pay attention to individual parts but didn’t care about the whole system, only pay attention to measurement of appearance but didn’t care about the essence of things. Those dialectical analyses provided a solid theoretical basis for the construction of a new psychological test system. Clearly, we must have a correct guiding ideology, focused on understanding and mastering the law of nature in doing research. In 1965, Prof. Cao wrote in a preface of translating foreign books, “If we engaged in psychological research without the correct philosophical thought as theoretical guidance, although sometimes you may get some specific results, but we could not get the right conclusions about the nature of the laws of psychology”; “on the basis of existing research results, the use of dialectical materialism will make the experimental study of psychology to a new level”.

Because of the conscious use of the dialectical materialism in research practice, Prof. Cao has made outstanding achievements in the field of experimental psychology, especially in learning and memory research. It still has a great impact on the development of memory psychology in current China.

From 1945 to 1948, Prof. Cao has been studying psychology at Cambridge University. His project focused on “The Time between Learning and Memory” (Zhao, 2001). During this time, he published many results on the experimental study and graphics memory recognition research in foreign journals. These results are now used as a model of early experimental study of learning and memory in China. In the early 1960’s, his work at Cambridge was used to study the effect of time interval on haptic. In his experiment, he was both the experimenter and subject. It was a very specific and unique design. In 1962, he guided a memory research group. On the one hand, he instructed the staff to study the effects of different sensory channels (visual, tactile and auditory) on memory and to explore the mental mechanism of memorization by Pavlov’s theory of advanced neural activity and information theory. On the other hand, he also combined his research with practical education. He studied junior middle school students’ memorizing methods of four-character phrases such as, classical Chinese essays. He did this while making a comparative analysis of the process of memorization of the classical and colloquial texts to improve teaching methods. Although his work was not able to be completed because of the Cultural Revolution, it is important to know that Prof. Cao created his own complete system of ideas on memory in psychological research. He regards memory as the process of encoding, storing and extracting input information. He has also systematically explained the different mechanisms of instantaneous memory, short-term memory and long-term memory. There is no doubt that he is the pioneer of information processing theory in China. Based on these long-term accumulated research projects and learning achievements, the general psychology course edited by Prof. Cao has become the enlightening material of many well-known psychologists. This book is widely circulated in China and has been reprinted more than 20 times!

In addition, Prof. Cao has contributed to the development of science and industry for our country. He served as the Planning Bureau, Liaison Office Deputy Director of Chinese Academy of Sciences (CAS), Deputy Director and Director of Institute of Psychology, CAS. In the early days of new China, he spared no effort to help drifters in a foreign country sent back to China, which included the famous scientist Tsien Hsueshen (Xuesen Qian, 钱学森). In the meanwhile, Prof. Cao is responsible for the preparation of the Institute of Psychology of CAS and the Chinese Psychological Society. He devotes all his life for the construction of psychology in our country. He put dialectic materialism in the research of psychology as he has mentioned in his later years, “In the work of psychological research, we should be upheld the guide line to carry out theory with practice, serve the socialist construction. Research topics should be satisfied to the needs of socialist construction, and the needs to solve the psychological problem in socialist construction.” This study, which analyzes attitudes and philosophical thought, is not only the requirement for his research work, but also the inspiration for future researchers. It enlightens us to use dialectical methodology to seriously explore and solve scientific problems.
